# Primary healthcare providers challenged during the COVID-19 pandemic: a qualitative study

**DOI:** 10.1186/s12875-022-01923-4

**Published:** 2022-12-03

**Authors:** Lusine Aslanyan, Zaruhi Arakelyan, Astghik Atanyan, Arpine Abrahamyan, Manya Karapetyan, Serine Sahakyan

**Affiliations:** Turpanjian College of Health Sciences, American University of Armenia, 40 Marshal Baghramian Ave, 0019 Yerevan, Armenia

**Keywords:** Primary healthcare, COVID-19, Providers, Risk communication, Supply distribution, PPE, Specimen collection, Home visits, Patients, Qualitative research

## Abstract

**Background:**

Primary healthcare (PHC) providers are widely acknowledged for putting the most efficient and long-lasting efforts for addressing community health issues and promoting health equity. This study aimed to explore PHC providers’ experiences with coronavirus pandemic preparedness and response in Armenia.

**Methods:**

We applied a qualitative study design using semi-structured in-depth interviews and structured observation checklists. Study participants were recruited using theoretical and convenience sampling techniques throughout Armenia. Inductive conventional content analysis was utilized to analyze the in-depth interviews. Nineteen in-depth interviews were conducted with 21 participants. Observations took place in 35 PHC facilities. The data collected during the observations was analyzed using the “SPSS22.0.0.0” software.

**Results:**

Five main themes of primary healthcare providers’ experiences were drawn out based on the study findings: 1) the gap in providers’ risk communication skills; 2) uneven supply distributions; 3) difficulties in specimen collection and testing processes; 4) providers challenged by home visits; 5) poor patient-provider relationships.

The results revealed that primary care providers were affected by uneven supply distribution throughout the country. The lack of proper laboratory settings and issues with specimen collection were challenges shaping the providers’ experiences during the pandemic. The study highlighted the health systems’ unpreparedness to engage providers in home visits for COVID-19 patients. The findings suggested that it was more challenging for healthcare providers to gain the trust of their patients during the pandemic. The study results also underlined the need for trainings to help primary care providers enhance their risk communication expertise or assign other responsible bodies to carry out risk communication on PHC providers’ behalf.

**Conclusion:**

The study discovered that PHC providers have a very important role in healthcare system’s preparedness and response to handle public health emergencies such as the COVID-19 pandemic. Based on the findings the study team recommends prioritizing rural PHC development, ensuring appropriate supply distributions, developing comprehensive protocols on safe home visits and specimen collection and testing processes, and trainings PHC providers on risk communication, patient-centeredness, as well as proper use of personal protective equipment.

**Supplementary Information:**

The online version contains supplementary material available at 10.1186/s12875-022-01923-4.

## Introduction

The coronavirus disease (COVID-19) [1] pandemic has been defined as a global health crisis, causing major challenges for the health systems worldwide [2]. The World Health Organization (WHO) expressed concerns, particularly for nations with underdeveloped healthcare systems, highlighting the need of bolstering the health systems' front lines, particularly primary care [3]. Given its capacity to lessen the burden on hospitals, serve as a gateway for patients to secondary and tertiary care, and significantly contribute to the achievement of health equity and universal health coverage during the crisis, primary healthcare (PHC) has played a determining role during the COVID-19 pandemic [4–7].

Globally, since the onset of the COVID-19 pandemic, PHC services have undergone a rapid shift to better serve patients with and without COVID-19, with an emphasis on patient and healthcare worker safety [4–7]. The involvement of PHC services in the detection, clinical management, and follow-up of COVID-19 patients has drastically altered the scope of operations, capacity, and function of PHC services. PHC has also been the key player in the delivery of mass vaccinations. In the meantime, changes in the management of non-COVID-19 patients, provision of essential health services, and methods of risk communication also took place in PHC facilities [6, 8, 9].

In several studies, the effects and difficulties of PHC system reform during the COVID-19 pandemic were examined in the context of the experiences of health professionals. For instance, on-the-ground consultations in primary care were gradually partially replaced by remote consultations using telephone calls and telemedicine[10–12]. Studies have documented both the advantages and disadvantages of this shift: while telemedicine allowed for greater flexibility and patient-centered care, it also increased the workload for PHC providers and created uncertainty in their decision-making regarding care prioritization, which in some cases raised ethical questions [11–13]. During the first wave of the pandemic, home visitation units were reduced in many countries in order to minimize the danger of virus transmission, restricting consultations to urgent care only [14–16]. However, home delivery of medications was also widely practiced [11, 17]. A few studies emphasized the difficulties PHC professionals had faced as a result of adjustments made in reaction to the pandemic, including a tremendous workload that was difficult to manage, an increase in the burden of administrative duties, and low job satisfaction [6, 16–19]. According to other studies, PHC providers struggled to integrate to new workflows because of lack of resources and training [20–22]. In some limited resource settings, insufficient PHC facility preparedness and lack of equipment were also documented, causing poor working conditions which resulted in reduced quality of care and increased risk for both patients’ and health workers’ safety [14, 19, 21, 23].

Along with strengthening the PHC, effective risk communication to healthcare professionals and the general public is another crucial aspect of the pandemic response. This includes messages on how to deal with misinformation, deception, and the resulting psychological strain, as well as information on preventative actions for harm reduction and preventing the spread of the disease [24, 25]. Previous studies have shown how crucial it is for the government and healthcare organizations to offer and disseminate accurate, timely, and educational health risk information [26, 27].

In Armenia, national response to COVID-19 started in March, 2020. On March 16, the government declared a three-month state of emergency to control the spread of infection in the country. The main measures against the spread of COVID-19 included mask wearing, social distancing, quarantine and isolation, along with dissemination of health messages and risk communication to raise the general public’s awareness on COVID-19 and its prevention. At the beginning of the outbreak, testing and treatment services were available at designated hospitals and National Center for Disease Control laboratory. Starting from May, 2020, testing (sample collection and transportation to designated laboratories) and outpatient care for patients with COVID-19 were expanded to PHC facilities and private health facilties [28].

In Armenia, PHC sector involves 352 public and 148 private facilities and other PHC units that provide state-guaranteed health services to over 98% of the population [29]. Services provided include immunizations; screening and diagnostic services; specialist consultations; chronic disease management; maternal and child health services; home visits and others [30–32]. PHC supply procurement is usually organized at the local level and only medication is procured centrally by the national government. With the start of the pandemic a few changes were made to the procurement procedures ensuring adequate supply distribution throughout the PHC facilities. Legal revisions were introduced to ensure accelerated supply acquisition and distribution, as well as more funds were allocated for supply purchasing [33].

In the light of the COVID-19, preparedness of the health system for the future pandemics largely depends on adequate and informed planning of operations. Hence, knowledge of challenges and limitations in the performance of health system and its infrastructures is of utmost importance for informed decision-making. Additionally, since PHC physicians are often the initial point of contact for patients visiting both private and public PHC facilities, they can provide the general public with useful insights into "what works and what doesn't". Moreover, in many countries, including Armenia, PHC providers became responsible for specimen collection, testing, and provision of initial care to COVID-19 patients [28].

Despite the large number of qualitative research on the PHC preparedness for the COVID-19 pandemic, less information is available on investigating the experiences of primary care providers during the pandemic in terms of response to COVID-19 in particular nations or areas. Thus, the study team aimed to explore PHC providers (general practitioners and family physicians) experiences in the preparedness and response of PHC to COVID-19 pandemic in Armenia.

## Methods

### Study design

We applied a qualitative study design using semi-structured in-depth interviews and a structured observation checklist to explore primary healthcare providers’ experiences during the pandemic. The rationale for conducting qualitative research was to address our study aim of exploring healthcare providers’ experiences more in-depth.

### Study settings, participants and sampling

We recruited study participants by using theoretical [34] and convenience [35] sampling throughout Armenia including the capital city (Yerevan) and provinces (Ararat, Syunik, Tavush, Aragatsotn, Shirak, Armavir). The theoretical sampling included analyzing data during the data collection process to decide further types of professionals we might need to interview and what type of additional data we should collect.

As part of the convenience sampling technique we approached the PHC providers through the administration of the corresponding PHC facilities. The rest of the participants were contacted directly through the social/professional network of the research team. We recruited PHC providers (general practitioners and family physicians) from public and private PHC facilities involved in diagnosis and treatment of COVID-19 patients. In Armenia, general practitioners and family physicians are part of the PHC workforce. General practitioners usually work in urban facilities and serve adult population only and family physicians usually work in rural facilities [32]. We also recruited policy makers, PHC facility managers as well as patients who had a COVID-19 diagnosis and either received or did not receive services from PHC facilities.

We conducted observations in PHC facilities of Armenia, both in the capital city and provincial facilities. Proportionate to size random sampling was implemented to select 36 urban PHC facilities in Yerevan (*n* = 13) and provinces (*n* = 23). The study included only urban facilities for the observation purposes considering feasibility issues.

### Study instruments

The research team reviewed the local and international scientific evidence, guidelines, standard operating procedures and recommendations on COVID-19 regulations in primary healthcare facilities to develop the study instruments: the interview guides and observation checklist. In-depth interview guides were developed specifically targeting each category of the study participants: the PHC providers, patients and policy makers (Appendices 1, 2 and 3). The guides contained open-ended questions on the main themes, each followed by probing questions to allow collection of in-depth information from the study participants about their experiences with providing/receiving PHC services during COVID-19. The main domains of the interview guide used with the PHC providers were risk communication (RC), availability of appropriate resources to ensure proper provision of services, specimen collection, testing practices, and case management. The guide targeting patients mainly included questions regarding their experiences during specimen collection and how they were managed during their disease by their PHC providers or other healthcare providers. The policy makers’ guide targeted questions regarding PHC system’s preparedness and response to the COVID-19 pandemic and areas for improvements.

The study team finalized the observation instrument (Appendix 4) after discussion with an expert epidemiologist. It consisted of two sections: observation of the facility common areas, including healthcare providers’ protective behavior, and a standardized checklist on supply availability and distribution of those in the facility.

The observation checklist and the interview guides were initially developed in English, then translated into Armenian. We piloted the observation checklist in one of the PHC facilities. Based on the experiences of the pilot, the research team improved the flow and the formulation of the checklist items. The interview guides were continuously refined as part of the theoretical sampling.

### Data collection, management and analysis

The research team conducted data collection activities from May to September 2021. We conducted nineteen in-depth interviews with a total of 21 participants -9 PHC providers, 10 patients and 2 policy makers. As part of the theoretical sampling technique we continuously refined the interview guides during the data collection process to cover newly developed themes. Data collection stopped at meaning saturation [36, 37], which was identified through simultaneous data collection and analysis, and data collection from each category of respondents was stopped when further interviews were not able to generate any new information.

Four independent researchers from the study team conducted most of the in-depth interviews remotely utilizing different virtual platforms such as Zoom. All of the remote calls were video-assisted to foster rapport building. Following the participants’ priority, seven interviews were conducted face-to-face, keeping social distance and using N95 respirators. Interviews were audio- recorded getting permission from the study participants. If a study participant refused to be audio recorded, the moderator only took notes during the interview. The mean duration of in-depth interviews was approximately 43 min, ranging from 30 to 69 min. The interviewers also collected information about the participants’ age, gender and place of residence. They also asked the PHC providers if they worked at a public or a private polyclinic.

The interviewers themselves transcribed and analyzed recordings and notes in the original language. Then the representative quotes selected for the paper were translated into English. We used inductive conventional content analysis [38] to analyze the transcripts. The collected data was coded by words and meaningful sentences that were later grouped into several categories. The categories were further grouped under subthemes. Some of the themes were developed based on the discussions with interviewees that were not incorporated in the instrument. All of the themes explored wide range of differences from the perspective of urban and rural communities.

One of the researchers conducted the visits to all chosen polyclinics for the observations through the IPC standardized checklist. The observation took place in 35 PHC facilities, out of which 3 were private and 32 were public. The researcher conducted the observation with a help of a tablet in the “Alchemer” portal. The observation was conducted through interviews with the head of the PHC facility and two PHC providers per facility as well as through observing the behavior of the PHC providers and filling out the relevant checklist.

The data collected data during the observation was exported from the “Alchemer” portal in SPSS format, cleaned and analyzed through “SPSS 22.0.0.0” software. The checklist gave an opportunity to compare the supply distributions from the perspective of the head of the polyclinic and the perspective of the PHC providers. It also allowed the research team to look at supply distributions in Yerevan versus the provinces.

### Study rigor

To build rapport between the interviewers and participants and to ensure credible responses, trained and experienced researchers with relevant background conducted the interviews with each group of participants: a healthcare provider with a public health background interviewed the PHC providers and a social worker with a public health background interviewed the patients, and a public health specialist with the policy makers.

Frequent peer-briefing meetings took place to discuss the data collection and analysis process improving the trustworthiness [39] of the research. The interviewers also conducted member checking to improve the rigor of the research. Transcripts were sent back to participants for member checking to remove inaccurate information. Interviewers applied this technique for all in-depth interviews. The research team ensured the credibility [39] of the study by conducting interviews in different regions of Armenia, including both urban and rural areas, and engaging three different groups of stakeholders with different perspectives in the study. We collected data through several methods (in-depth interviews and observations), which allowed methodological triangulation [40].

## Results

### Participant demographics

The recruited participants were from Yerevan (*n* = 7), as well as Syunik (*n* = 5), Tavush (*n* = 3), Aragatsotn (*n* = 1), Ararat (*n* = 3), Armavir (*n* = 1); and Shirak (*n* = 1) provinces. We had four male and 17 female participants. The mean age of the participants was 47 years ranging from 23 to 64. The number of patients was 10. The number of the PHC providers were nine eight of which worked at a public polyclinic and one in a private (Table 1).

**Table 1:** Characteristics of participants

Participant categories	Number of participants by gender (n)			Mean age (years)	Primary healthcare facility type		Number of participants by study site (n)	
	**Male**	**Female**	**Total**		**Private**	**Public**	**Urban**	**Rural**
PHC providers	1	8	9	53	1	8	4	5
COVID-19 patients	3	7	10	47	-		10	0
Policy makers	-	2	2	50	-		2	-

### Themes

The data suggested five themes. The gap in providers’ risk communication skills theme explores PHC providers’ involvement in the risk communication management with the community, their satisfaction regarding implemented strategies, and self-perception and involvement in those activities. The uneven supply distributions theme tells the level of preparedness with the necessary equipment for personal protection combining findings from the interviews and observation. The difficulties in specimen collection and testing processes theme presents results on the differences in challenges of assigning patients to testing laboratories for COVID-19 in rural vs urban areas. The providers challenged by home visits theme investigates the challenges related to home visits by the PHC providers given the restricted resources and patient adherence to home visits regulations. The final theme, patient-provider relationships, introduces dissimilarities of patient-provider relationships in urban and rural areas.

### Gap in providers’ risk communication skills

The study results demonstrated providers’ limited perception of their own role and responsibility in risk communication with communities. Their perception of RC entailed raising awareness among patients only when they reached out and asked questions.

*“It happens, we were not directly told [to spread information], but the population visits us themselves [with the questions].” PHC provider, Female, Province.*

According to the interviewed physicians there was lack of training and preparation on how to conduct effective communication with public. Despite the fact that there were online seminars regarding COVID-19, none of these seminars covered how the information should be delivered to the community and patients.

*“…nobody involved us in it [in seminars regarding RC], hence we couldn’t take part.” PHC provider, Female, Yerevan.*

The participants felt the need of the specific seminars about proper information dissemination and sharing skills development. Some of the providers noted that RC shouldn’t be included in their responsibilities given their overloaded schedules. They recommended that other specialists should take charge of that.

*“…for that purpose (RC) there should be a separate specialist…. Obviously, there should be a program, people who will get salary, will go and explain the steps to those people (community members) and how they (community members) should do it.” PHC provider, Female, Yerevan.*

The most crucial difference observed in terms of RC was the response of rural healthcare providers to the needs of community. Overall, both the majority of rural and urban participants did not see any specific actions they could have undertaken in RC process, however more commonly rural providers were ready to organize, help and lead. A participant mentioned that they were working closely with the local municipality and organizing awareness raising activities:

“*In our [name of the medical center] we were implementing awareness raising strategies. The community was always in touch with us, as well as the municipality employees, they also did a lot in terms of spreading [information] from their end…” PHC provider, Female, Province.*

One of the PHC providers recruited a young woman from her community to spread qualified and “*evidence-based*” information among the same community. As the recruited woman was very “*active*” and “*well known*” among the habitants, awareness level was increased within the community.

### Uneven supply distributions

Supply distribution was noted to be one of the challenges in terms of providing quality healthcare services to the community not only by the PHC providers but the policy makers as well.

*“The burden brought by COVID was huge [in terms of supply distributions]. There were already problems before COVID, COVID made them worse.” Policy maker, Female, Yerevan.*

One of the most noticeable differences was the presence of supply shortages in Yerevan’s public PHC facilities, whereas in rural areas the only challenge was the delay of supply distribution. The supply shortages in Yerevan were highly relevant during the beginning of the pandemic, especially in the public facilities.

*“We got nothing, they should have distributed, but our facility did not provide us with anything. We even bought our goggles ourselves; they are just now starting to distribute something. Not even gloves.” PHC provider, Female, Yerevan.*

The majority of healthcare providers from rural areas mentioned that sometimes there were delays of supply distribution because of which the healthcare workers were buying the supplies themselves, but eventually the PHC facility was equipped with the necessary supplies.

*“We obtained [supplies] by ourselves, then we received [from the government]. At some point we realized very few is left, then we bought again…it was more convenient for me that way, instead of waiting until the government will obtain and send to me, that would have been too late.” PHC provider, Female, Province.*

Besides minor delays of supply in rural settings, the PHCs here were provided with appropriate supplies by charitable organizations as well. Many were provided with such type of supply even before the pandemic.

*“[Names a charitable organization] also provided us with coats, hats. We use them until now, they are really good ones.” PHC provider, Female, Province.*

According to additional file 1 the total percentage of facilitates providing personal protective equipment (PPE) to their healthcare providers from the perspective of the facility heads’ were the following for these certain types of PPE: surgical masks – 100%, respirators—77%, gowns – 97%, gloves – 100%, goggles – 97%, face shields – 100%. Respirators (54%) and goggles (77%) availability largely differed when considering the PHC providers’ perspective. When asked about if the facilitates provide certain types of PPEs to the PHC providers in sufficient quantities, the total percentages were the following according to the facility heads: surgical masks – 86%, respirators – 67%, gowns – 82%, gloves 86%, goggles – 91% and face shields – 100%. Notably, all these percentages are lower when compared to the percentages of facilities providing PPE (not necessarily in sufficient quantities) to the healthcare workers. The total percentages of facilities providing PPE in sufficient quantities were somewhat different (either higher or lower) for certain types of PPEs when considering the perspectives of PHC providers (Additional file 1).

There were a few notable differences when comparing the percentages of facilities providing PPE to PHC providers in Yerevan vs the provinces. These numbers were markedly different when considering the perspectives of facility heads about respirators: 62% in Yerevan and 86% in provinces. The percentages of facilities providing PPE to PHC workers in sufficient quantities were also somewhat different for certain PPE supplies when looking at the differences between Yerevan and provinces based on both facility heads’ and providers’ prospective (Additional file 1).

Another interesting finding from the observation that confirmed the findings from the in-depth interviews was the behavior of the observed PHC providers in terms of wearing masks. During the observation, the mean percentage of observed PHC providers wearing masks in Yerevan facilities was 52% with the highest percentage being 80% and the lowest 19%. In provinces, the mean percentage of PHC providers wearing masks was 36% with the highest percentage being 100%. The lowest percentage was 0%, meaning in some facilitates healthcare providers did not wear masks at all.

### Difficulties in specimen collection and testing processes

A difference noted during discussions was the challenges in rural areas compared to the urban regarding specimen collection. In rural areas providers were sometimes unable to test patients due to small number of laboratories, absence of laboratories in rural areas, absence of transport, shortage of fuel or low number of tests (transportation to the nearest laboratory was done only in case of fixed number of specimens).

*“There is such a problem here. We collect the specimen, but the laboratory is in Ijevan [Ijevan is the province center and they live further from] do you see that car? It was provided to us last year … no fuel, nothing, if you can manage to make it work, do it.” PHC provider, Male, Province.*

In some cases, doctors did not have any other choice then to ask the patient to take their own specimen to the nearest laboratory.

*“There is a problem with budget… why should the member of my community take their specimen to Ijevan…., or some of them agree to take their specimens together, so that it won’t be expensive for them.” PHC provider, Male, Province.*

In urban settings the only challenge regarding this topic were waiting lines in the laboratories:

*“Well obviously we did not wait in the polyclinic [to get tested], as we did not trust them as much and it was the season with the highest peak [of cases] with enormous waiting lines…I went and paid to get tested not to lose any time.” Patient, Male, Yerevan.*

None of the urban PHC providers mentioned any challenges regarding testing process, some of the interviewed participants even mentioned that they did not know the process after specimen collection, as the nurse is generally taking care of it.

*“We take it [the specimen], fill in everything, attach everything and the nurse takes it.” PHC provider, Female, Yerevan.*

### Providers challenged by home visits

This theme was discussed from different perspectives: PHC providers, patients and policy makers. A major gap was noticed during discussions with providers in terms of differences of home visits in the urban vs rural areas. In the rural areas most of the PHC facilities had only one family physician, unlike the facilities in the cities. Hence if the only physician of the facility got infected with COVID during the home visit, that could have had enormous negative impact on the functionality of the facility. Another common opinion regarding home visits was voiced by 2 of the participants. They told that in rural settings, the patient may ask for home visit but when the physician got there, there was a chance that the patient would be out doing their routine village work.

*“In terms of home visits to COVID-19 confirmed patients, if there is only one general practitioner in that PHC facility or community, you should keep that doctor safe, that is my personal opinion. If the nurses are trained, they go, they check the temperature, and they check the overall well-being.” PHC provider, Female, Province.*

Throughout the discussions some of the participants also mentioned that the habitants of rural settings very often do not take into consideration that the working day has finished and they may call for home visits even at night.

*“Usually, the concept of home visits is a little bit out in the air. In practice it’s not the same. You go to the home visit, the house owner [the patient)] is in the garden [working], the house owner (the patient) took the animals to pasture.” PHC provider, Male, Province.*

Policy makers stressed about the importance of home visits and that it was challenging to ensure proper and “*uninterrupted home visits*”: They also reflected on the organizational flow and the challenges to address technical issues such as transportation and proper PPE supply for the PHC providers during home visits:

*“[We couldn’t ensure] things like uninterrupted availability of transportation, so that the team [PHC providers] could go [do the home visits]. [We couldn’t ensure] [PHC providers] to be protected, so that everything would have been safe for them.” Policy maker, Female, Yerevan.*

### Patient-provider relationships

According to the findings there were two categories of patients who “*did not trust*” their healthcare provider. In one case, there was an absence of trust that the provider genuinely cared for patients’ interests, was honest, practiced confidentiality, and had the competence to produce the best possible results. The participants said that they faced difficulties in establishing good doctor-patient communications, which made them find someone else to monitor the whole treatment process. Patients sought a quick resolution to their ailments by using their personal network and frequently calling several physicians to obtain a satisfying answer.

*“By the way, I am very dissatisfied with the attitude of the doctor of that polyclinic. Well, as a pregnant woman, at least they should have helped me in a special way, right? At least they should have done an X-ray, they should have been more careful as I am pregnant. I did not see any such approach from them at all. That's why I went to a paid hospital.” Patient, Female, Yerevan.*

The second category of patients was from provinces. They had good relationships with their healthcare providers or knew each other personally (regional communities are very small) but patients perceived them as “*less qualified*” compared with healthcare providers of Yerevan. Irrespective of their gratitude towards their providers they still sought the advice of other qualified healthcare providers from Yerevan and made their own decisions by using mixed treatment approaches.

*“I definitely obey the doctor of our polyclinic, but since we also have acquaintances—doctors, nurses among our close relatives and taking into account all that I listen to their advices, they worked in the ambulance during those crisis situations in our Armenia. It's very personal, especially if I have had surgery.” Patient, Female, Province.*

## Discussion

Given the scarcity of studies investigating healthcare providers’ experiences administering quality service provision at the PHC level during COVID-19, our study sought to fill this gap qualitatively exploring the factors challenging PHC providers work during the pandemic. The PHC preparedness and response to the COVID-19 pandemic was explored through the experiences of family physicians and general practitioners in both rural and urban areas of Armenia.

The study identified five themes underlying PHC providers’ experiences during the pandemic: gap in providers’ risk communication skills, uneven supply distributions, difficulties in specimen collection and testing processes, providers challenged by home visits and patient-provider relationships.

Risk communication is an important aspect of public health and its role is even more important during outbreak prevention and control [26]. Although RC has initially not been part of PHC provision in Armenia, considering its huge impact on PHC globally during the pandemic [26, 41–43], we would like to discuss a few significant discoveries concerning the PHC providers' RC abilities. The providers' comprehension of what RC entails was limited. In some cases, they acknowledged that their personal RC competence was insufficient and emphasized the need for either trainings to help them advance their RC knowledge and skills or for other responsible entities to undertake risk communication function on their behalf because of their busy schedules. These findings were in line with studies from China and Bangladesh [44, 45].

One of the important components of our study findings was related to the unequal distribution of PPE supplies throughout the country, despite the newly introduced legal changes that were meant to accelerate the supply procurement and distribution process [33]. This issue was especially obvious when comparing the capital city Yerevan and the provinces. While there were delays in the delivery of goods to the provinces, Yerevan's facilities frequently experienced a scarcity of materials. When contrasting the views of PHC providers and facility managers regarding supply problems, an intriguing difference was discovered. Compared to healthcare providers, the facility managers had a more optimistic uptake regarding the supplies in their facilities. When questioned about the same materials, PHC providers generally believed that they had fewer items and in smaller amounts than the facility heads had stated. The greatest shortage was reported about respiratory masks in all facilities we visited. These findings were consistent with previous studies examining essential IPC and PPE supplies, particularly facemasks crisis during the outbreak [46, 47].

Lack of adequate laboratory settings and other problems with specimen collection were explored through the experiences of healthcare providers and patients. The study findings revealed long waiting times in front of specimen collection locations being one of the biggest problems with laboratory testing in urban facilities. In rural settings, the PHC providers collided with the issue of appointing their patients’ specimens to laboratories given the limited laboratory sites in their region [48, 49]. These findings can also be explained by the fact that in Armenia before the pandemic, the laboratory facilities were mainly located in urban areas [50]. At the beginning of the COVID-19 pandemic, the specimen collection and testing processes in already existing laboratories were gradually extended involving more human and technical resources to respond to the pandemic [50]. However, the main focus still remained on the urban areas, unintentionally leaving rural areas out of the focus.

The results of our study provided information about providers' perceptions of home visits for COVID-19 patients. The health system’s preparedness to conduct home visits was noted to be insufficient. Providers were reluctant to visit their patients at home as their facilities lacked the means to ensure proper and effective personal protection. At the same time, most PHC providers avoided home visits based on their fear to get infected; hence, they switched to calling the patients instead of home visits [11, 21, 51]. The avoidance of home visits resulted in enhanced application of telemedicine (using telephones and other online platforms for patient care) which according to the literature could potentially result in higher flexibility but at the same time more workload [11–13].

Patient-provider relationships were the last key component uncovered by the study results. The main conclusion in relation to this issue was that patients found it difficult to develop a relationship of trust with their healthcare providers during the pandemic [52]. In Yerevan, the limited trust was mostly explained by the lack of communication skills of healthcare providers. Although patients in the provinces had better ties with their PHC providers, they still trusted more skilled specialists from Yerevan regarding COVID-19 [53]. Patient-provider relationships have generally been a core issue in the Armenian healthcare system. A study conducted to assess outpatient tuberculosis care in Armenia confirmed our findings and showed that education, psychiatric care, and family support should all be included in a more people-centered treatment strategy in primary healthcare [54].

There were several limitations in this study. As the study participants choose whether or not to participate, there was a chance of self-selection bias. However, the use of multiple data sources has minimized this bias. Some study participants may also have provided more socially desirable answers; hence, the real situation might be worse than described. Although the study team applied several measures (triangulation, member checking, collecting data through different methods and in different geographical areas) to enhance the rigor of the study. Researcher bias (related to correct interpretation of the findings) might still have influenced the results. To address this issue, frequent peer-briefing meetings took place to decrease potential researcher bias. Finally, the observation took place only in urban facilities due to feasibility limiting the generalizability of our findings.

## Conclusion

The study found that primary healthcare providers’ experiences were key to shape healthcare system preparedness in response to public health crisis situations such as the COVID-19 pandemic. The results of this study could help to come up with recommendations to improve the overall experiences of healthcare providers working in primary care settings during public health emergencies. Moreover, considering that the characteristics discussed as part of our study findings need to be addressed at the baseline level, the study results might have a key impact on an improved rapid response for future pandemics.

The study findings highlight the importance of developing a national comprehensive strategic plan for primary healthcare preparedness and response to future pandemics, using an equity-based approach towards urban and rural areas. The strategy will ensure prioritizing trainings among healthcare providers about the importance of risk communication, proper use of personal protective equipment, and patient-centered practices. The national plan should also emphasize an exhaustive plan ensuring proper supply distributions throughout the PHC facilitates across the country, improved access to specimen collection and laboratory testing as well as protocols for safe home visits.

## Supplementary Information

**Additional file 1.** 

**Additional file 2.**

## Data Availability

The datasets used and/or analyzed during the current study are available from the corresponding author on reasonable request.

## Abbreviations

COVID-19: Coronavirus disease 2019
WHO: World Health Organization
PHC: Primary healthcare
RC: Risk communication
PPE: Personal protective equipment
