# Supplementary material for: Primary healthcare providers challenged during the COVID-19 pandemic: a qualitative study
Source: BMC Prim Care. 2022 Dec 3;23:310. doi: 10.1186/s12875-022-01923-4 (PMC9719166; doi:10.1186/s12875-022-01923-4)
Supplement: Supplementary file 1 — Additional file 1. [file 12875_2022_1923_MOESM1_ESM.docx]

**Additional file 1: Availability of personal protective equipment (PPE) in the PHC facilities from the perspective of PHC facility heads and providers by Yerevan and provinces**

|  | **Surgical masks** | | **Respirators** | | **Gown** | | **Gloves** | | **Goggles** | | **Face shield** | |
| --- | --- | --- | --- | --- | --- | --- | --- | --- | --- | --- | --- | --- |
|  | Facilities providing PPE to PHC providers, N (%) | Facilities providing PPE to PHC providers in sufficient quantities  N (%) | Facilities providing PPE to PHC providers, N (%) | Facilities providing PPE to PHC providers in sufficient quantities  N (%) | Facilities providing PPE to PHC providers, N (%) | Facilities providing PPE to PHC providers in sufficient quantities  N (%) | Facilities providing PPE to PHC providers, N (%) | Facilities providing PPE to PHC providers in sufficient quantities  N (%) | Facilities providing PPE to PHC providers, N (%) | Facilities providing PPE to PHC providers in sufficient quantities  N (%) | Facilities providing PPE to PHC providers, N (%) | Facilities providing PPE to PHC providers in sufficient quantities  N (%) |
|  | **According to PHC facility heads** | | | | | | | | | | | |
| **Yerevan** | 13 (100%) | 10(76.9%) | 8(61.5%) | 6(75.0%) | 12(92.3%) | 10(83.3%) | 13 (100%) | 12(92.3%) | 13(100%) | 13(100%) | 13(100%) | 13(100%) |
| **Provinces** | 22(100%) | 20(90.0%) | 19(86.4%) | 12(63.7%) | 22 (100%) | 18(81.8%) | 22 (100%) | 18(81.8%) | 21(95.5%) | 18(85.7%) | 22(100%) | 22(100%) |
| **Total** | 35(100%) | 30(85.7%) | 27(77.1%) | 18(66.7%) | 34(97.1%) | 28(82.4%) | 35(100%) | 30(85.7%) | 34(97.1%) | 31(91.2%) | 35(100%) | 35(100%) |
|  | **According to PHC providers** | | | | | | | | | | | |
| **Yerevan** | 26(100%) | 24(96.0%) | 13(50%) | 13(100%) | 26(100%) | 26(100%) | 24(92.3%) | 25(100%) | 22(84.6%) | 23(100%) | 26(100%) | 26(100%) |
| **Provinces** | 42(95.5%) | 37(88.1%) | 25(56.8%) | 16(64.0%) | 43(97.7%) | 37(86.0%) | 42(95.5%) | 39(92.9%) | 32(72.7%) | 31(93.9%) | 42(93.2%) | 37(90.2%) |
| **Total** | 68(97.1%) | 61(91.0%) | 38(54.3%) | 29(76.3%) | 69(98.6%) | 63(91.3%) | 66(94.3%) | 64(95.5%) | 54(77.1%) | 54(96.4%) | 67(95.7%) | 63(94.0%) |
