# Supplementary material for: Primary healthcare providers challenged during the COVID-19 pandemic: a qualitative study
Source: BMC Prim Care. 2022 Dec 3;23:310. doi: 10.1186/s12875-022-01923-4 (PMC9719166; doi:10.1186/s12875-022-01923-4)
Supplement: Supplementary file 2 — Additional file 2. [file 12875_2022_1923_MOESM2_ESM.docx]

**Appendices**

*Appendix 1* – Primary Care Providers Interview Guide

**Primary care providers interview guide**

Hello and thank you for agreeing to participate in this interview. Please, tell us a little about your main role and activities in this facility. How much have your responsibilities been changed after the COVID-19 pandemic?

*Now let us talk about the extent of healthcare providers’ involvement in the risk communication with the community regarding the COVID-19.*

1. What can you say about the awareness-raising activities on COVID-19 in the country? For example, what do you think about the methods and communication channels that were used to inform the public.
2. How targeted, timely and continuous were they.
3. How clear and understandable were they for different groups of people (children, people with disabilities, elderly, etc.) What would you suggest for the future?
4. How do you perceive your role as a PHC provider in those activities? Have you been approached by the community with questions on the diseases? Have you been prepared to provide, educate the patients on this?
5. Have you been involved in any activity that aimed to discuss/develop what information needs to be provided to the public and how? If not, would you like to be involved in such an activity?

*Now I would like to discuss the following topics with you.*

1. *on-time preparedness, access, and provision with the information procedures, and tools required for self-protection and effective work.*

*b) the provided support and practiced self-coping mechanisms at the beginning and midst of the pandemic.*

1. How did you feel at the beginning of the outbreak, both as a person and as a health care provider? What did you do in response to the disease outbreak?
2. What can you say about your on-time preparedness? For example, did you have enough access, and provision with the needed information? How proactively have you communicated what is known, what is unknown?
3. Have you been informed about the procedures and tools required for self-protection and effective work? If yes, when and how have you been provided with this?
4. How and when you were provided with adequate PPE supplies and appropriate IPC to protect yourself as a health care provider? Have you been trained enough to know how to use PPE and IPC at the beginning and midst of the pandemic?
5. What can you say about your physical condition during the pandemic? Did you have time to get enough rest and food?
6. Had you been provided with any psychological or materialistic support during the pandemic to overcome the difficulties at work? For example, from colleagues, from the facility manager, government? If yes, how supportive they were?
7. What would you recommend for the future?

*Let’s discuss some questions regarding testing practices, particularly COVID-19 testing strategies and case notification practices at the PHC level*

1. What are the current testing strategies, i.e indications in case of certain symptoms, risk groups, or patient status (suspected case, contact, etc.). How much it was changed during the time. What do you think about the changes that happened during the time?
2. Is COVID-19 testing conducted in laboratories within the PHC level? If not, what testing referral system is in place? Where the specimen collection takes place? What alternative testing facilities and sites are available to assist in case finding (in communities)?
3. In case of a severe case when a patient is not able to go for testing, how do you manage the testing, diagnosis, and further treatment process? (Is there a special service that collects a specimen in these cases? Is it free of charge?)
4. How healthcare providers are being notified about changing testing strategies? What sources of information do the healthcare providers use to learn about the updates on testing strategies?

*The following questions are regarding home visits.*

1. How do you assess the need for a home visit? How do you do the home visit? Please describe a bit more about it.
2. How do you manage patients whose condition is deteriorated during off-hours or weekends?

Thank you for participating in the interview. Is there anything on this topic that you would like to add?

*Appendix 2* – Patients Interview Guide

**Patients Interview Guide**

Hello and thank you for agreeing to participate in this interview.

*Let us talk about your experience with catching the COVID -19 virus.*

1. When you suspected that you might have the COVID-19 virus, what were your next steps? Whom did you contact (polyclinic, hospital, private clinic) first?
2. If you contacted the polyclinic, how did they manage you as a suspected case? How did you communicate with the polyclinic before the testing? (probe: did you have a phone call, a visit, or the doctor visited you?).
3. If you visited the polyclinic, what was the reason for the visit? (probe: who instructed you to visit or was it a self-made decision).
4. How satisfied were you with the polyclinic services? What would you improve?
5. If the doctor visited you, what was the reason for the visit? Please tell us about the process of the home visit (probe: what did the doctor instruct and recommend about the treatment and how to protect family members)? How satisfied were you with the doctor’s home visit? What would you improve?

*Now let’s talk about the testing experience. Please, describe how it went?*

1. How did the polyclinic carry out (manage) the testing process? Where did you go for testing? How did the PHC providers inform you about the testing process and procedures required to follow before the testing? How did the polyclinic manage you as a suspected case while you were waiting for your test results?
2. Overall, how satisfied were you with the testing process and the quality of tests used? What would you improve?

*Shall we talk about your treatment now? Could you please describe your experience with your treatment? How did it go?*

1. After confirming your test results, how did your doctor manage you as a confirmed COVID-19 case? How did you keep contact with the polyclinic/doctor (how often)?
2. What were your main concerns during your disease and the treatment? How would you recommend improving the polyclinic’s role in managing COVID-19 cases?
3. If you were not hospitalized, did you think you should have been and why?

*I would like to ask you several questions given that you haven’t approached the polyclinic to manage your case [ask this question only to those patients who haven’t approached the polyclinic for their treatment].*

1. When you suspected that you might have the COVID-19 virus, whom did you contact (hospital, private clinic, private polyclinic, etc) first? Could you tell us why was the polyclinic not your first to-go choice? Why did not you contact them or your primary healthcare provider?
2. How and where were you tested?
3. After confirming the test results, what were your next steps? Please tell us about the process of your treatment and the medication used: how did you get treated? For how long? Did you undergo additional diagnostic tests? How did you know that you recovered and can go back to your everyday life?

*I would like to ask you a few questions to summarize our conversation.*

1. Please tell us about your overall impression of your experience with COVID-19. What are your main concerns now that you have been treated against the virus? What would you recommend to improve in the healthcare system during the fight against the COVID-19 virus?

*Appendix 3* – Policy Makers Interview Guide

**Policy Makers Interview Guide**

Hello and thank you for agreeing to participate in this interview.

1. Please tell us briefly about your current and previous position/positions (if it has changed in the last year and a half), your areas of responsibility and how the COVID-19 pandemic has affected your work.
2. In your opinion, how did Armenia's healthcare system in general cope with the pandemic (for example, in terms of system readiness, human resources and capabilities, property and technical adequacy)? What do you think could have been done better?
3. Now I would like to ask the same question, but about the Armenian government in general. In your opinion, what did the government do well/or continues to do well in relation to the pandemic? What can be done better? What does the government need to be better prepared for similar situations in the future (additional resources, different procedures, removal of existing barriers)?
4. Please describe what changes have occurred in the PHC sector due to the COVID-19 pandemic. How have reallocations of resources been made shifting from day-to-day extensive services to essential/priority services?
5. What communication approaches were used to build public trust and encourage continued use of essential/priority services during the outbreak (for example, through polyclinics, pharmacies, community health workers and managers)?
6. What changes have occurred in the salaries of employees? Have there been changes to the payment schedule (delays)? Did the staff work overtime? Is there adequate compensation for overtime work?

*Appendix 4* – Observation standardized checklist instrument

**Facility standardized checklist and observation form**

| **No.** | **Questions** | **Response options** |
| --- | --- | --- |
|  | Interviewer/Observer ID |  |
|  | Facility ID |  |
|  | Managing authority | 1. State 2. Private |
|  | Date of the observation | ______/_______/________  *dd mm yyyy* |
| **5.** | Role of the respondent(s) in the facility | Facility manager/epidemiologist (please underline)/other (please specify below)  _________________________ |

**Please complete the following section with the help of facility manager/epidemiologist or other dedicated personnel (specify____________).**

| **No.** | **Questions** | **Response options** | | **Comments** |
| --- | --- | --- | --- | --- |
| **Personal Protective Equipment (PPE)** | | | | |
| **6.** | The facility provides PPE to health workers. | Yes ☐ | No ☐ |  |
| **7.** | The following items are currently available for each of the staff who are required to use them in accordance with the applicable guidelines: (Please ask the question about sufficient quantity of each PPE in case of its availability) |  |  |  |
| **7.1** | - Protective gown | Yes ☐ | No ☐ |  |
| **7.1.1** | protective gown is in sufficient quantity | Yes ☐ | No ☐ |  |
| **7.2** | - Mask, medical/surgical | Yes ☐ | No ☐ |  |
| **7.2.1** | medical/surgical masks is in sufficient quantity | Yes ☐ | No ☐ |  |
| **7.3** | - Gloves | Yes ☐ | No ☐ |  |
| **7.3.1** | Gloves are in sufficient quantity | Yes ☐ | No ☐ |  |
| **7.4** | - Goggles | Yes ☐ | No ☐ |  |
| **7.4.1** | Goggles are in sufficient quantity | Yes ☐ | No ☐ |  |
| **7.5** | - Face shield | Yes ☐ | No ☐ |  |
| **7.5.1** | Face shields is in sufficient quantity | Yes ☐ | No ☐ |  |
| **7.6** | - Respirator masks (N95 or FFP2) | Yes ☐ | No ☐ |  |
| **7.6.1** | Respirator masks are in sufficient quantity | Yes ☐ | No ☐ |  |
| **8.** | The facility has a stable supply | Yes ☐ | No ☐ |  |
| **8.1** | - PPE for patient care | Yes ☐ | No ☐ |  |
| **8.2** | - Other essential supplies (e.g., ABHR/hand sanitizer, liquid soap, and paper towels) | Yes ☐ | No ☐ |  |
| **9.** | Does the facility have an SOP for safe disposal of the used PPE. | Yes ☐ | No ☐ |  |
| **10.** | What additional equipment/services/supplies does your facility need to better respond and prevent COVID-19 (specify)?   ___________________________ |  |  |  |

**Please fill the following section by conducting an observation of the reception and/or waiting areas (especially around the general practitioners’ office) of the facility. Spend 1 hour in the reception and/or waiting areas at the beginning of the working day to observe.**

| **No.** | **Questions** |  |  | **Comments** |
| --- | --- | --- | --- | --- |
| **IPC instructions** | | | | |
| **Hand hygiene performance** | | | | |
| **11.** | There is an access to alcohol-based hand rub (ABHR)/hand sanitizer with alcohol in the following areas: |  |  |  |
|  | - At the entrance | Yes ☐ | No ☐ |  |
|  | - Waiting areas | Yes ☐ | No ☐ |  |
| **12.** | Please observe for 15 min if people entering the facility (including healthcare providers, other staff, patients and visitors) perform hand hygiene upon entering the primary care facility: |  |  |  |
|  | - How many people have entered the facility? | **__________** | |  |
|  | - How many of them performed hand hygiene? | **__________** | |  |
| **Respiratory hygiene performance**  ***Please observe for 15 min the medical staff’s and patients/visitors respiratory hygiene compliance at the reception and/or waiting.*** | | | | |
|  | - How many staff did you observe? | **__________** | |  |
|  | - How many of them were wearing mask? | **__________** | |  |
|  | - How many patients/visitors did you observe? | **__________** | |  |
|  | - How many of them were wearing mask? | **__________** | |  |

**The following questions should be completed through an interview, with general practitioners/family physicians.**

|  | I would like to ask you to think about essential clinical equipment and supplies, computer equipment, and software you usually utilize while working with COVID-19 patients.  Now I will ask you some questions about them to assess their availability and needs for quality care of COVID-19 patients. | | | | |
| --- | --- | --- | --- | --- | --- |
| **13.** | Are the following equipment available for your everyday use? If yes, are those in sufficient amounts? | | | | |
|  | - Equipment | Availability | | Sufficient quantity | |
|  | - Protective gown | Yes ☐ | No ☐ | Yes ☐ | No ☐ |
|  | - Mask, medical/surgical | Yes ☐ | No ☐ | Yes ☐ | No ☐ |
|  | - Gloves | Yes ☐ | No ☐ | Yes ☐ | No ☐ |
|  | - Goggles | Yes ☐ | No ☐ | Yes ☐ | No ☐ |
|  | - Face shield | Yes ☐ | No ☐ | Yes ☐ | No ☐ |
|  | - Respirator masks (N95 or FFP2) | Yes ☐ | No ☐ | Yes ☐ | No ☐ |
| **14.** | - What additional equipment/services/supplies does your facility need to better respond and prevent COVID-19 (specify)? |  |  |  |  |
